# Three-Dimensional Finite-Element Study into the Effect of Scar Tissue on the Displacement of the Maxillary Segments by Rapid Palatal Expansion in Unilateral Cleft Lip, Alveolus, and Palate

**DOI:** 10.1055/s-0045-1810017

**Published:** 2025-07-18

**Authors:** Irene Sanita Lanny, Cendrawasih Andusyana Farmasyanti, Anne Marie Kuijpers-Jagtman, Paramita Noviasari, Ananto Ali Alhasyimi

**Affiliations:** 1 Department of Orthodontics, Faculty of Dentistry, Universitas Gadjah Mada, Yogyakarta, Indonesia; 2 Department of Orthodontics, University Medical Centre Groningen (UMCG), Groningen, The Netherlands; 3 Department of Orthodontics and Dentofacial Orthopedics, School of Dental Medicine/Medical Faculty, University of Bern, Bern, Switzerland; 4 Faculty of Dentistry, Universitas Gadjah Mada, Yogyakarta, Indonesia

**Keywords:** rapid palatal expander, finite-element analysis, scar, unilateral cleft lip and palate, medicine

## Abstract

**Objectives:**

Scar tissue tension may potentially influence the effectiveness of rapid palatal expansion (RPE) in patients with unilateral cleft lip and palate (UCLP). Comprehending the biomechanics of expansion appliances in individuals with UCLP is crucial in determining the suitable RPE design to rectify the asymmetric arch. This study used finite-element analysis (FEA) to investigate how scar contractures affect maxillary segments displacement during RPE in UCLP patients.

**Materials and Methods:**

A three-dimensional model of the maxilla was constructed from cone beam computed tomography images of an 11-year-old boy with UCLP who received RPE. ANSYS 2022 R2 software was employed to conduct FEA simulations with this model. Three forces were considered: expansion force, upper lip tension, and scar tension. Two simulation conditions were analyzed: a control scenario with only the expansion force and a second scenario with all three forces applied. Displacement was assessed in transversal (x-axis), vertical (y-axis), and sagittal (z-axis) directions at 14 reference points located across the palate and dentition.

**Results:**

Both simulations exhibited similar patterns of maxillary displacement, but the magnitude of displacement was reduced when scar and lip tension were included. The minor segment exhibited a greater displacement than the major segment, with the largest movement occurring in the anterior region along the x- and z-axes, which gradually decreased posteriorly. The displacement pattern was as follows: x-axis > z-axis > y-axis for the major segment and z-axis > x-axis > y-axis for the minor segment.

**Conclusion:**

The FEA model demonstrated that RPE in UCLP produces an asymmetrical expansion with a pyramid-shaped displacement pattern. However, when scar tension from the palate and the upper lip is included, the extent of the segmental movement is reduced. These findings suggest that scar tissue tension may potentially influence the effectiveness of RPE in patients with UCLP.

## Introduction

One of the most prevalent congenital defects is unilateral cleft lip and palate (UCLP) with a prevalence of 1.37 per 1,000 births in low- and middle-income countries. The majority of UCLPs are nonsyndromic. 1 2 The maxillary tissues are divided by this condition into a major and a minor segment. 3 Postnatal surgical repair is an essential treatment; however, it may result in significant soft tissue tension caused by scar contractures. 4 The lack of elasticity, dense collagen fibers, and low concentration of hyaluronic acid result in rigidity of hypertrophic scars. 5 The combination of an intrinsic maxillary growth deficiency, functional disturbances due to disturbed muscle function, and the presence of scar tissue following cleft repair is responsible for maxillary growth deficiency in the sagittal, transverse, and vertical directions, along with a marked crossbite, in patients with a cleft. 4 6 7 Therefore, maxillary expansion therapy is frequently employed in orthodontic treatment for patients with UCLP. 8

Maxillary expansion with a rapid palatal expansion (RPE) plays a crucial role in the management of the arch width constriction improving palate volume, enabling additional space for appropriate tongue posture, and producing proper arch alignment prior to alveolar bone grafting. 7 8 9 The expansion appliances included tooth-borne, hybrid, and bone-borne expanders. RPE as a tooth-borne device is a well-documented tool for maxillary expansion in patients with UCLP. 10 The Hyrax expander is the most commonly used type of RPE because it is comfortable and hygienic, causes minimal irritation, and does not worsen speech difficulties in patients with a cleft. 11 *In silico* studies have shown that applying a 5-N force through RPE can produce skeletal and dental expansion in UCLP. However, these studies did not account for the presence of scar tissue of the palate and upper lip. 12 13 Al-Gunaid et al 14 and Kim et al 15 suggested that scar tissue tension in the palate and upper lip might exert an inward opposing force, potentially affecting the expansion force of the RPE. Nevertheless, there remains a lack of detailed research into how scar tissue and upper lip forces specifically RPE outcomes in patients with UCLP.

*In vivo* observation of tissue responses to RPE forces in patients with cleft lip and palate is challenging because load transfer to the alveolar process and scar tissue forces cannot be measured directly. Finite-element analysis (FEA) has become a valuable tool for studying orthodontic biomechanics. 16 17 This approach enables a comprehensive examination of the biomechanical effects of procedures such as RPE, which offers a better understanding of the distribution of stress and deformation within these biological tissues. This facilitates better treatment planning while minimizing the risk of adverse effects. 18 However, its application in cleft lip and palate research has been limited. 19 A previous FEA study examined the effects of post–cleft repair scars and maxillary arch expansion, combined with maxillary protraction therapy, on maxillary development. The study found significant growth restrictions primarily in the transverse and sagittal directions. 20 However, that study did not specify the type of expander appliance used or the exact region of interest (ROI). Moreover, the study used upper lip pressure values from individuals without a lip scar and did not account for the forces exerted by scar tissue, which are believed to affect the expansion biomechanics.

Understanding the biomechanics of expansion appliances in patients with UCLP is important for selecting the appropriate RPE design to correct the asymmetric arch. Therefore, our study aims to address this gap by using FEA to study displacement patterns during RPE treatment in patients with UCLP. We will consider the influence of scar contracture following reconstructive surgery for UCLP, focusing on displacements along the transverse (x-axis), vertical (y-axis), and sagittal (z-axis) directions in defined ROI. This study hypothesizes that the tension of scar tissue on the palate and upper lip restricts maxillary segmental displacement during RPE in patients with UCLP.

## Material and Methods

### Preprocessing Stage

**Fig. 1:**
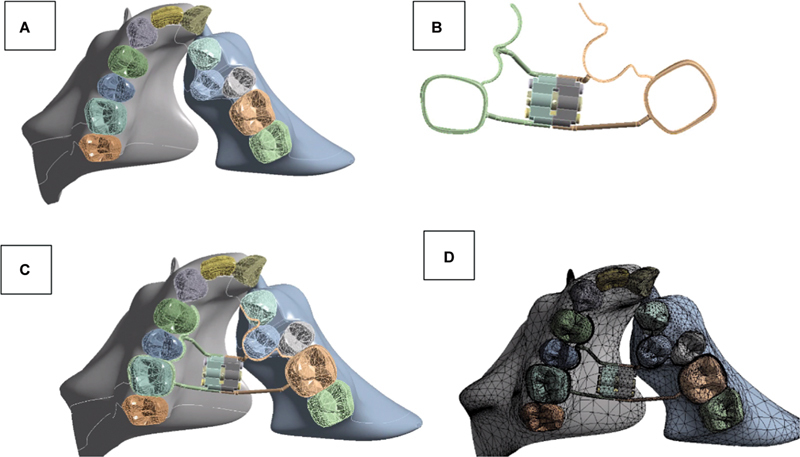
Three-dimensional (3D) model. ( **A** ) 3D maxilla model, ( **B** ) 3D Hyrax expander model, ( **C** ) 3D simulation model, and ( **D** ) mesh of the 3D simulation model.

Ethical clearance was granted by the ethics committee of the Faculty of Dentistry, UGM (No. 71/UN1/KEP/FKG-RSGM/EC/2023). A finite-element model was derived from a cone beam computed tomography (CBCT) image of a 11-year-old boy with UCLP who was operated for the cleft of the lip and palate, and now planned for alveolar bone grafting. Written informed consent was obtained from the parents. The 3D model of the maxilla was created digitally using Autodesk Inventor Professional 2019 (Autodesk Inc, San Rafael, CA, United States). A 3D model of the Hyrax palatal expander was created based on its specifications (Hyrax Mini-7, Dentaurum, Ispringen, Germany; Fig. 1 ). The placement of the 3D RPE model onto the 3D maxillary model was performed using the same software. Subsequently, the combined model was imported into the ANSYS 2022 R2 software (ANSYS Inc, Canonsburg, PA, United States) to conduct meshing. The meshing process generated 384.154 nodes and 223.979 elements. Mesh convergence experiments were conducted prior to conducting any structural analysis to ensure that the discretization model (the mesh) selected accurately represents the structure without introducing substantial errors. This procedure included the development of numerous meshes with varying densities and the subsequent analysis of the results to confirm that the predicted outcomes are no longer significantly affected by further mesh refinement. The convergence test was conducted by analyzing models with increasing mesh densities (element counts: 50,000, 100,000, and 150,000). Displacement and stress values showed less than 2% variation between the two finest meshes, indicating numerical convergence and confirming that the FEA results are both accurate and reliable.

All structural materials were categorized as isotropic, linear, elastic, and homogenous. The cortical and trabecular bone were considered as a single, homogeneous material in order to minimize computational expenses. This assumption may influence the quantitative stress and strain analysis. To reduce this error, the values of Young's modulus and Poisson's ratio were calculated for the bone and teeth ( Table 1 ). The mechanical properties, including Young's modulus and Poisson's ratio, were to provide values for calculation. These parameters define the natural properties of the constructed model, enabling it to function and respond similarly to a natural biological system when exposed to external stimuli (stress).

**Table 1:** Young's modulus and Poisson's ratio of various materials used in this study

Structure	Young's modulus (MPa)	Poisson's ratio
Rapid palatal expander 20 21	200,000	0.33
Bone 20	10,000	0.30
Teeth 20	20,000	0.30
Scar tissue 20 22	24.22	0.50
Upper lip 23	0.5	0.50

### Processing Stage

Boundary conditions were implemented to enforce symmetry conditions. The posterior regions were designated as fixed in all directions due to the rigidity imposed by the skull base. The expansion force was applied to the palatal surface of the upper first premolar and first molar, with the force vector aligned parallel to the transverse plane. The simulations could then be performed after the assignment of material properties and boundary conditions. The simulation included application of three forces: expansion force, upper lip tissue tension, and palatal scar tissue tension ( Table 2 ). To simulate daily clinical expansion, expanders were activated transversely to produce 0.25-mm displacement at the level of the expansion screw. Two simulation scenarios were analyzed. The first served as a control, only applying an expansion force. In the second, all forces were applied. In the latter, the same expansion force was applied as in the first simulation, along with a 3.7-N upper lip scar force applied perpendicular to the anterior surface of the alveolar process at the vestibular level 23 and a 17.52-N palatal scar force from the scar edge to the center of the scar tissue. 22

**Table 2:** Setting of the working conditions

Force name	Force area	Force value (N)	Force direction
Expansion force 20 21	Palatal surface of the upper first premolar and first molar	5	Parallel to the transverse plane
Lip force 23	Anterior process alveolaris from canine to canine at the vestibulum level	3.7	Perpendicular to the anterior surface of the alveolar process
Scar force 22	Scar tissue on the mid-palatal	17.52	Along the scar, from outside to inside radially

### Postprocessing Stage

**Fig. 2:**
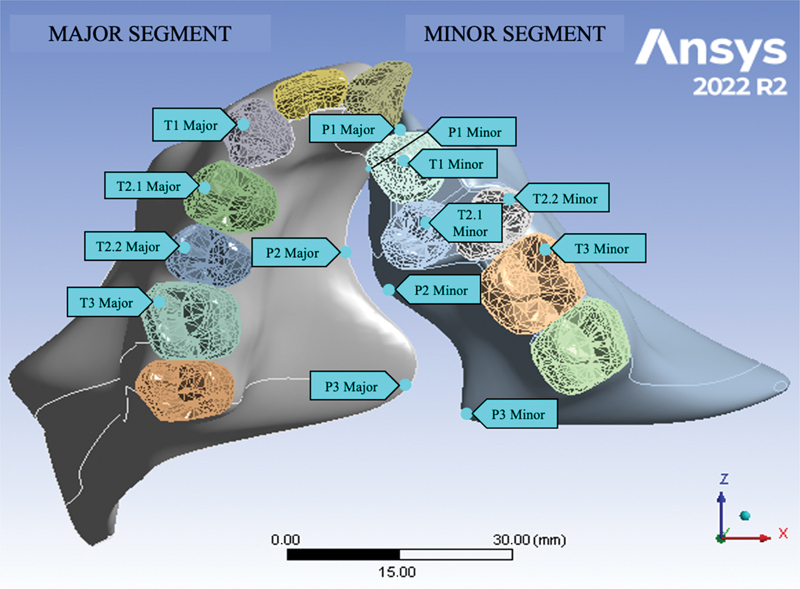
Evaluating landmarks.

The final stage involved the evaluation of the displacement pattern. The available data for analysis are presented numerically (in millimeters) and visually. This study examined 14 reference points along the palate and teeth on the major and minor segment in the x-axis (transversal), y-axis (vertical), and z-axis (sagittal; Fig. 2 ). The displacement values of the first and second simulations were evaluated at reference points on the palate and teeth of both segments representing the anterior (P1 and T1), middle (P2, T2.1, and T2.2), and posterior (P3 and T3) regions ( Fig. 2 ). Positive values indicate outward minor segment, upward, and forward displacements in the X, Y, and Z planes, respectively.

## Results

### Displacement Patterns: Visual

**Fig. 3:**
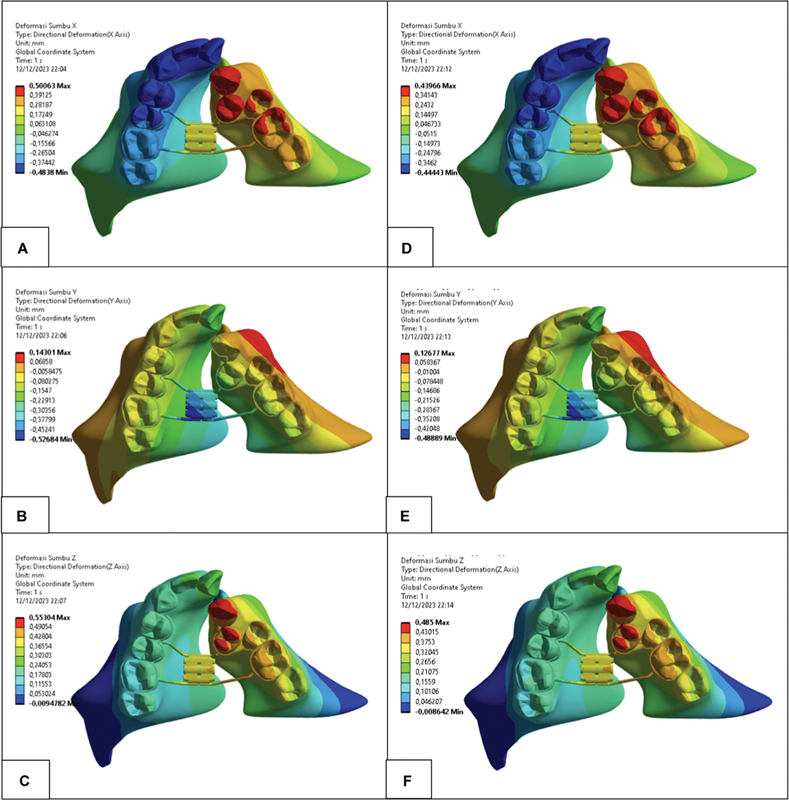
Displacement pattern: ( **A** ) expansion of the RPE on the x-axis, ( **B** ) expansion of the RPE on the y-axis, ( **C** ) expansion of the RPE on the z-axis, ( **D** ) combined with scar tissue on the x-axis, ( **E** ) combined with scar tissue on the y-axis, and ( **F** ) combined with scar tissue on the z-axis.

Data were evaluated both visually and numerically on each reference point of the ROI, which were on the anterior, middle, and posterior. Visually, the displacement pattern in Fig. 3 shows that the color contours are nearly identical between the simulation of the RPE expansion force alone ( Fig. 3a–c ) and the combination with scar tissue ( Fig. 3d–f ). The most significant displacement, shown in red, was observed in the minor segment. Transversally, it was most evident on the canine and first premolar as well as on the mesial side of the second premolar and first molar. Vertically, displacement occurred, but this is not within the observable region. Sagittally, it was visible on the canine and partially on the first premolar in the minor segment.

### Displacement Pattern of Applying a 5-N Expansion Force to the RPE

The results of the displacement caused by the RPE expansion force on the x-axis, y-axis, and z-axis of the major and minor segments are presented in Table 3 . Typically, the minor segment displacement is larger than that of the major segment, except on the y-axis. The displacement pattern of the major segments from largest to smallest was x-axis > z-axis > y-axis, which indicates that the maxilla has lateral widening, followed by forward and downward movements. The movement pattern of the minor segments from the largest to the smallest was z-axis > x-axis > y-axis, which indicates that the maxilla is moving forward, followed by lateral widening and downward.

**Table 3:** Displacements (mm) in the palate and teeth after applying expansion force RPE only and combined with scar and upper lip force

Reference point	Simulation 1: only the expansion force						Simulation 2: expansion force combined with scar and upper lip forces					
	Major segment			Minor segment			Major segment			Minor segment		
	x	y	z	x	y	z	X	y	z	x	y	z
**Palate**												
P1	− 0.3501	− 0.2046	0.2401	0.4431	− 0.0214	0.404	− 0.3224	− 0.1963	0.215	0.3266	− 0.0212	0.3421
P2	− 0.1937	− 0.2154	0.1826	0.2028	− 0.2289	0.3131	− 0.1782	− 0.2022	0.1622	0.1731	− 0.2027	0.2754
P3	− 0.099	− 0.3627	0.2107	0.1248	− 0.3053	0.2855	− 0.0948	− 0.3449	0.1871	0.1092	− 0.2766	0.2513
**Teeth**												
T1	− 0.4714	− 0.0354	0.2171	0.5006	− 0.0277	0.553	− 0.435	− 0.0329	0.1986	0.4397	− 0.0248	0.485
T2.1	− 0.4421	− 0.0196	0.198	0.4497	− 0.0804	0.5109	− 0.4137	− 0.0179	0.1822	0.3969	− 0.0712	0.4491
T2.2	− 0.4112	− 0.0209	0.1815	0.4223	0.0592	0.4394	− 0.3829	− 0.0192	0.1678	0.3711	0.0529	0.3874
T3	− 0.3704	− 0.0245	0.1656	0.4134	0.0252	0.4382	− 0.3413	− 0.0226	0.1543	0.3675	0.0321	0.3864
Mean	− 0.334	− 0.1262	0.1994	0.3652	− 0.1068	0.4206	− 0.3098	− 0.1194	0.181	0.312	− 0.0974	0.3681

Note: The x-axis refers to transverse displacement: (+) cleft expansion and (−) noncleft expansion. The y-axis refers to the vertical displacement: (+) upward and (−) downward. The z-axis refers to sagittal displacement: (+) forward.

Transversally, on the x-axis, the largest displacement values were found at P1 and T1 in both segments, which decreased gradually posteriorly. Vertically on the y-axis, the value was in the P3 major segment and T2.1 minor segment; a pattern could not be determined. Sagittally, on the z-axis, the largest displacement values were found anteriorly (represented by P1 and T1), which decreased gradually toward the posterior.

### Displacement Pattern of Applying a 5-N Expansion Force to the RPE Combined with Palatal Scar Tissue and Upper Lip Forces

The results of the displacement caused by the RPE expansion force combined with palatal scar tissue and upper lip forces on the x-axis, y-axis, and z-axis of the major and minor segments are presented in Table 3 . Transversally, on the x-axis, the largest displacement values were found at P1 and T1 in both segments, which decreased gradually posteriorly. Vertically, on the y-axis, the largest displacement values were in P3 major and T2.1 minor segments, a pattern could not be determined. Sagittally, on the z-axis, the largest displacement values were found anteriorly (represented by P1 and T1), which decreased gradually toward the posterior.

Maxillary displacement in the first and second simulations produced the same pattern; however, the displacement value decreased in the second simulation. The minor segment showed a greater decrease in displacement values, particularly in the x- and z-axes. The greatest decrease occurs in the anterior segment, which progressively diminishes toward the posterior segment.

## Discussion

The FEA is a proven mathematical tool for noninvasive evaluation of orthodontic biomechanics and its effects on the craniofacial complex. 24 FEA allows for the simulation of clinical orthodontic scenarios by adjusting force magnitude, direction, and point of application, enabling the calculation of stress experienced at any specific point. 25 The biomechanical mechanism and effects of FEA are highly dependent on the quality of the constructed models used to simulate the real structure, which can be influenced by the number of elements. Our maxillary simulation model was created manually using the nonuniform rational basis spline (NURBS) method because the CBCT and scanner scan results obtained were surface models. Consequently, if the model is sliced across, the central section of the model will be devoid of any material, and microsections cannot be comprehended during the meshing process. The creation of NURBS curves begins by determining the number and position of curves that represent anatomical reference points from the original object to produce a solid model with more optimal mesh quality. 26 In this study, the 3D finite-element model comprised 384.154 nodes and 223.979 elements, which exceeded those of other related studies. 12 27

RPE is known for using heavy forces to obtain skeletal treatment effects. In patients with UCLP, a force of 5 N can achieve orthopedic expansion. 12 28 One turn (90 degrees) on a screw is equivalent to a widening of 0.25 mm, which produces a force of 6.8 to 8.8 N, and approximately 85% of the force is received by the palate and anchor teeth. 29 30 Asymmetric expansion may occur because of differences in segment resistance; the minor segment has less resistance than the major segment, resulting in greater lateral expansion as was also found in our study. 30 The minor segment expansion force is composed of an outward force to expand the arch and a downward and forward rotating force of the maxilla to correct the minor segment into a more ideal arch relationship. 14 This is in accordance with the movement of the y- and z-axes in both simulations, which shows the direction of the downward and forward maxillary dislocation.

In the major segment, a different pattern was observed: the maxilla moved more laterally, forward, and then downward. This is in accordance with the expansion pattern of the RPE in the transverse plane, that is, the two parts of the maxilla will be separated triangularly, with the apex near the frontomaxillary suture and the base in the alveolar region. 7 Sagittally, the RPE produces forward movement of the maxilla with a tendency for the maxilla to reposition vertically downward. 11 The largest displacement values were found in the transverse and sagittal dimensions in the anterior region, which gradually decreased toward the posterior region. These findings support previous clinical studies in which Hyrax-type expanders had a triangular pattern with a wider base in the anterior region, representing 55% of the total expansion at the level of the first upper premolars, 45% at the second upper premolars, and 38% at the first upper molars. 31 Furthermore, Liu et al 32 emphasized the use of 2D measurement methodologies to evaluate the mid-palatal suture following rapid maxillary expansion procedures. The palatine suture's anterior expansion ranges from 2.42 to 4 mm (34.6–50% of the total screw expansion) and from 0.84 to 2.88 mm (12–36% of the total screw expansion) in the posterior region. Clinically, the greater expansion in the anterior region could be explained by the resistance of the medial and lateral pterygoid plates of the sphenoid bone to maxillary displacement during RPE expansion. 11 Another feasible explanation is that the direction of the expansion force produced by the expanders would be located anterior to the center of resistance of each maxillary half. 33

Maxillary displacement in the first (only expansion force) and second (expansion force plus upper lip tissue and scar tissue tension) simulations produced the same pattern; however, the displacement value decreased in the second simulation. This finding shows that the RPE produces sufficient expansion force despite the opposing force from scar tissue during the expansion. A heavier force is not necessary for maxillary expansion in UCLP because there is no mid-palatal suture and hence the resistance from the mid-palatal suture is negligible. 8 12 This suggests that standard RPE protocols may still be effective in UCLP cases without requiring force intensification, potentially minimizing the risk of adverse effects.

This study is the first to utilize FEA to examine the biomechanics of RPE expansion in UCLP, specifically considering the impact of scar tissue on the palate and in the upper lip. Understanding the biomechanics of RPE in UCLP cases, particularly in the cases where scar tissue is present along the dental arch, is important for several reasons. First, it provides insight into the mechanical behavior of the maxilla under expansion forces, which can help orthodontists predict and mitigate potential complications associated with scar tissue. This knowledge can direct the choice of suitable device designs and the RPE activation procedure, ensuring more effective treatment outcomes. Second, by understanding the displacement patterns and stress distributions in the clefted maxilla, clinicians may be able to customize their expansion devices. As the presence of scar tissue in patients with orofacial clefts can influence the pattern and likelihood of orthodontic relapse, the findings from this FEA study may also serve as a basis for further research, potentially leading to the development of surgical and orthodontic techniques that better accommodate the unique challenges presented by patients operated for an orofacial cleft. Palatal tension, scar tissue, and inadequacies in hard and soft tissues in UCLP cases lead to relapse and instability, rendering it more complex than in noncleft patients. The relapse and instability rates were statistically significantly correlated with the amount of bone resorption. 10

Nevertheless, this study has several limitations that must be acknowledged. First, the establishment of the model was based on a single patient with UCLP, which limits the ability of the model to accurately simulate the diverse clinical deformations observed in craniofacial structures across different patients. Second, this FEA study, similar to others, is limited by the mathematical models and assumptions used, which may not entirely reflect the complexities of the clinical scenarios. Moreover, the simplified material characteristics of craniofacial tissues in the model were neither homogenous nor elastic, which affected the simulation outcomes of deformation. Typical complications of RPE, such as buccal tipping of the anchor teeth, 7 11 were not observed because the model treated the palate and teeth as a single unit. This simplification overlooks significant factors like the musculature attached to the maxillary and circumaxillary bones, soft tissue fascia, periodontal ligaments, dentition, and dentoalveolar bone, all of which influence RPE outcomes. Future research could incorporate model validation against experimental data or clinical observations to increase the accuracy of model predictions. Experimental research on suture characteristics could provide more precise outcomes, and a mechanobiological assessment might enhance the understanding of bone remodeling. This could yield valuable clinical insights and help improve treatment methods for better patient care. While FEA provide a valuable foundation for understanding the effects of force application on various models, the limitations outlined here underscore the need for more comprehensive and clinically validated approaches in future research.

## Conclusion

The FEA model demonstrated that RPE in UCLP produces an asymmetrical expansion with a pyramid-shaped displacement pattern. However, when scar tension from the palate and the upper lip is included, the extent of the segmental movement is reduced. These findings suggest that scar tissue tension may potentially influence the effectiveness of RPE in patients with UCLP. This insight can specifically inform the selection and design of expansion devices, guide activation protocols, and potentially minimize the risk of treatment relapse. By integrating biomechanical insights into clinical planning, the study establishes a basis for more individualized and effective orthodontic treatments in patients with UCLP.
